# The +Gz-induced loss of consciousness curve

**DOI:** 10.1186/2046-7648-2-19

**Published:** 2013-06-06

**Authors:** Typ Whinnery, Estrella M Forster

**Affiliations:** 1 Oklahoma City, OK 73142, USA; 2 Mustang, OK, 73064, USA

**Keywords:** Neurophysiology, Ischemia, Syncope, Acceleration, Consciousness, Unconsciousness

## Abstract

**Background:**

+Gz-time tolerance curves were developed to predict when exposure to +Gz stress exceeds human tolerance resulting in neurologic signs and symptoms. The +Gz-induced loss of consciousness (G-LOC) curves were developed to predict when +Gz stress induces G-LOC. The G-LOC curves are based on a theoretical understanding of how acceleration affects underlying physiological mechanisms affording tolerance to acceleration, their limits, and what happens when they are exceeded. The foundation of previous +Gz-time tolerance curves was based on a minimal dataset of sign and symptom endpoints.

**Methods:**

Two G-LOC curves were established from the analysis of 888 centrifuge induced G-LOC episodes in completely healthy humans. The time from the onset of +Gz stress to the onset of unconsciousness was plotted as a function of +Gz level and the G onset rate.

**Results:**

The two new G-LOC curves differed significantly from previous curves in temporal characteristics and key aspects underlying neurologic response to acceleration. The new acceleration onset rate curve reveals that for onset rates ≥ 1.0 G/s, G-LOC will occur in a mean time of 9.10 s and is independent of the onset rate. The new +Gz-level curve demonstrates that G-LOC will occur in a mean time of 9.65 s for rapid onset rate exposures to +Gz levels ≥ +7 Gz. The minimum +Gz-level threshold tolerance was defined as +4.7 Gz. When +Gz onset rates are gradual, ≤ 0.2 G/s, G-LOC occurs in a mean time of 74.41 s. G-LOC did not occur earlier than 5 s for any acceleration exposure.

**Conclusions:**

These G-LOC curves alter previous temporal predictions for loss of consciousness and advance the understanding of basic neurophysiological function during exposure to the extremes of acceleration stress. Understanding the acceleration kinetics of the loss and recovery of consciousness provides the characteristics of uncomplicated and purely ischemic causes of LOC for application in medical diagnosis of syncope, epilepsy, and other clinical causes of transient loss of consciousness. The curves are applicable to education, training, medical evaluation, and aerospace operations.

## Background

It is well established that individuals exposed to acceleration (+Gz) stress beyond their tolerance level will lose consciousness. Current understanding of the cause of the loss of consciousness (LOC) is based on acceleration producing an environment in which the cardiovascular system is unable to supply an adequate amount of oxygenated blood to the cephalic nervous system (CPNS) regions that support conscious function. When the specific neurologic areas responsible for supporting consciousness are functionally compromised, LOC occurs. Fluctuations in cardiovascular function (heart rate, blood pressure, and vascular tone) occur regularly in normal environments, but are usually not of sufficient magnitude or duration to induce neurologic functional compromise. The nervous system has a buffer period that allows transient ischemia to be tolerated without symptoms or signs occurring. Fluctuations also occur in the acceleration (+Gz) forces humans experience in the Earth's gravitational field daily, beginning as one arises (horizontal to vertical) from sleep. Fortunately, it is only when acceleration stress applied in a specific direction, over a long enough duration, and at a sufficiently high magnitude, that neurologic function is embarrassed. Tolerance to acceleration is therefore defined by the stress envelope that will induce LOC. A clear description of the human acceleration tolerance envelope is therefore a component of understanding who we are and what environments we are able to safely enter without the threat of LOC.

Aircrew tolerance to the operational +Gz acceleration envelope of aircraft has been of concern from the beginnings of flight specifically as it related to +Gz-induced loss of consciousness (G-LOC) [1]. The adverse signs and symptoms associated with exceeding +Gz tolerance compromise flight performance and safety. Thus, aeromedical research programs were developed to understand and improve acceleration tolerance. An excellent description of the components of acceleration tolerance was developed by Burton [2-6]. Early acceleration research utilized general G-time tolerance curves integrating the relationship of +Gz level, time, and acceleration onset rate to understand and define the effects (symptoms and signs) of +Gz acceleration [7-10]. General +Gz-time tolerance curves define the envelope, separating states where an individual will be asymptomatic from those in which an individual will be symptomatic (visual, auditory, and conscious functional compromise). The upper limit of general +Gz-time tolerance curves is defined by +Gz-induced loss of consciousness. G-LOC is the major operational threat associated with exceeding +Gz tolerance, resulting in loss of aircraft and aircrew [11-16]. The definition of the G-LOC curve separating conscious and unconscious states is therefore a critically important aspect of the human response to acceleration, the development of +Gz-protective technology, and operational flight safety programs [17]. This study pursued development of the G-LOC curves that define the upper tolerance envelope of the +Gz level, exposure time, and onset rate in healthy humans.

The physiologic alteration induced by acceleration underlying loss of consciousness is considered to be the critical reduction of perfusion to cephalic regions of the nervous system for a specific period of time. These areas of the nervous system have the capacity to tolerate inadequate energy supply resulting from altered perfusion for a short period of time. The functional buffer period (FBP) is the time that the integrated neurologic function supporting consciousness is maintained following the loss of adequate energy necessary for sustaining normal function [18]. Noninvasively, the FBP can be determined as a component of the loss of consciousness induction time (LOCINDTI), the period that consciousness will be maintained when exposed to an acceleration profile that results in LOC. Experimentally, LOCINDTI is measured from the onset of acceleration to the onset of LOC and is an important component of G-LOC curves.

Previous +Gz-tolerance curves have been developed; however, the G-LOC aspect of those curves was based on a very limited number of actual LOC data points. One of the most frequently relied upon +Gz-tolerance curves was developed by Stoll as published in 1956 [19]. Inspection of the original publication reveals there were a total of 14 LOC data points, all of which were components of the left side of the +Gz-level-versus-time curve of her Figure three [19]. Stoll's +Gz-time curve was critically reviewed by Moore et al. [20] with the development of a mathematical G-time tolerance model. They concluded that improper joining of the data from different profiles produced an artifact - a dip in the horizontal (+Gz level tolerance) portion of the curve. Their tolerance curve described a hyperbola without any indication of a dip related to cardiovascular response time. They did not comment on the shape suggested by the Stoll curve or the continuously decreasing value of the curve's *y*-asymptote up to levels of +18 Gz with onset rates above 6 to 8 G/s.

Accurate, more operationally applicable G-time tolerance curves are needed [21]. An essential aspect of accurate G-LOC curves and one that applies to operationally important rapid onset profiles is the use of adequate data describing the LOCINDTI associated with the rapid onset acceleration (left side) arm of the curves. With very rapid onset and high +Gz profiles that exceed individual +Gz tolerance, the only defense to prevent LOC is the short time period the brain tolerates ischemia, the FBP. Based on 888 completely healthy human G-LOC episodes, mostly in experienced military fighter pilots, two G-LOC curves (+Gz level and +Gz onset rate versus time) were developed, recognizing these desirable objectives.

## Methods

### Subjects

This study was conducted utilizing G-LOC data retrieved from a centrifuge data repository describing the response of completely healthy humans to acceleration stress in a human centrifuge. The data are from volunteer research subjects, aircrew undergoing training to improve G tolerance [13], students in various aerospace medical disciplines, and aircrew undergoing medical evaluation [22]. All individuals successfully completed military physical examinations or the equivalent, with many having additional medical evaluation procedures to ensure normal health.

The data from the centrifuge repository contained 888 G-LOC episodes spanning the years 1978 to 1992, generated from centrifuge exposures at the USAF School of Aerospace Medicine, Brooks AFB, Texas and the Naval Air Warfare Center, Warminster, Pennsylvania. The repository did not identify the individual experiencing the G-LOC episode; therefore, the total number of individual subjects was approximately 723. There were 585 individuals having 1 G-LOC episode (585 episodes), 111 subjects experiencing two G-LOC episodes (222 episodes), and 27 experiencing three episodes (81 episodes), for a total of 888 G-LOC episodes.

### Procedures

For this study, the data included all G-LOC episodes that occurred. There was no separation of relaxed subjects, subjects performing an anti-G straining maneuver (AGSM), subjects wearing or without anti-G suits (AGS), or other combinations of anti-G protection methods or devices. The data therefore represented all +Gz exposures of healthy subjects who experienced G-LOC during various types of +Gz profiles. A variety of acceleration profiles existed in the data repository: (1) gradual onset rate (GOR), ramp (linear rise) profiles, (2) rapid onset rate (ROR), ramp to plateau profiles, (3) simulated aerial combat maneuvering profiles, (4) closed loop-centrifuge target tracking profiles, and (5) other complex experimental profiles. A large majority of the ROR profiles were a ramp to predetermined plateau resulting in G-LOC. The GOR profiles were predominately a linearly rising ramp to G-LOC. All subjects were in an upright sitting position (seat back angles 15° to 30° from the vertical) and maintained in that position with shoulder harness-lap belt restraint systems throughout the exposure including recovery of consciousness.

The methods used to describe G-LOC episodes and the methods for data collection and entry into the databases have been previously described [22,23]. All runs were terminated immediately upon recognition of G-LOC. G-LOC was identified (defined) by sudden muscle relaxation (facial, extremities, and torso), loss of postural tone, loss of response, loss of performance of a required task, and/or abrupt change in facial expression (eye fixation, staring, blank expression). No adverse events were associated with any of the G-LOC episodes. All G-LOC episodes, as recorded on videotape, were analyzed by at least two investigators, with agreement of all measurements of G-LOC onset to within 1 s. LOCINDTI was determined as the time period from base +Gz departure to the onset of G-LOC [22]. The onset rate (G/s) was calculated from the time and +Gz levels associated with the profile as the change in +Gz from base +Gz to the +Gz level when G-LOC occurred divided by the time. All profiles included in the G-LOC analysis began from a base +Gz level of ≤+1.7 Gz. The modern human centrifuges utilized in this study generated rapid onset rate profiles initiated from a rotating, low base +Gz level. The rapid onset profiles briskly approached the maximum instantaneous onset rates desired. Maximum instantaneous onset rates differed from the calculated onset rates by not more than ±0.1 G/s over the majority of ranges investigated up to 4 G/s. All experimental human research exposure to acceleration was approved by the advisory committees for human research at the respective institutions where the research was conducted: Naval Air Warfare Center (Advisory Committee for Human Experimentation) and USAF School of Aerospace Medicine (Committee for the Protection of Human Subjects). Data obtained from required military training were not attributable and did not require experimental consent.

### Statistical analysis

Descriptive statistics reported include mean, minimum, maximum, standard deviation (SD), standard error (SE), range, and median. Comparison analysis was conducted using analysis of variance (ANOVA) and *t* test statistics. Post-hoc analysis was conducted using Tukey's honest significant difference (HSD). Statistical significance was established at alpha = 0.05. Standard curve fitting methodology was utilized to determine the best fit, including iterative least squares analysis for optimal curve delineation.

## Results

**Figure 1:**
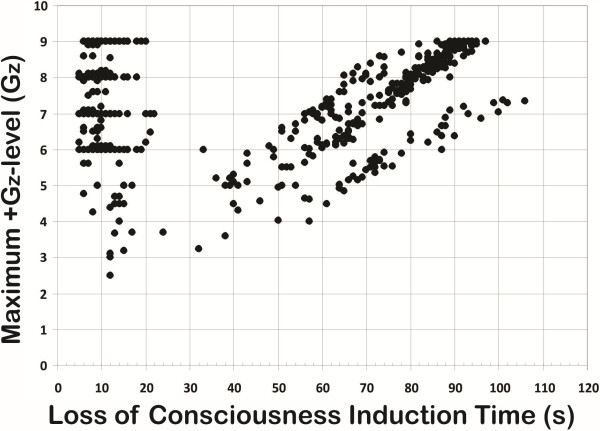
Maximum +Gz at LOC versus LOCINDTI for 749 G-LOC episodes.

**Figure 2:**
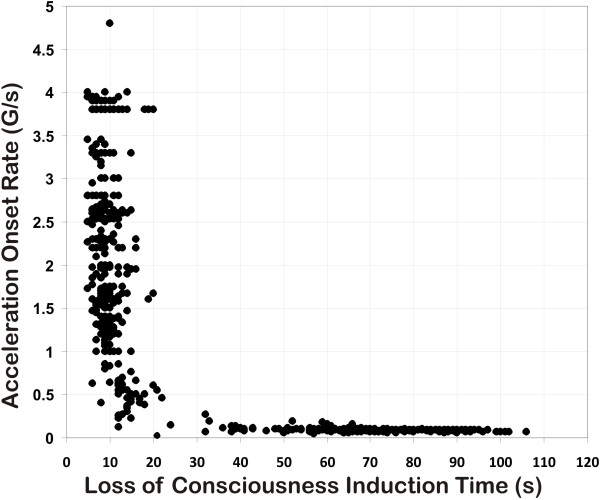
Acceleration onset rate versus LOCINDTI for 729 G-LOC episodes.

The experimental data points for the two G-LOC curves are plotted in Figures 1 and 2. Figure 1 describes the relationship of the +Gz level of exposure to LOCINDTI (the maximum +Gz level curve). Figure 2 describes the relationship of the onset rate to LOCINDTI (the acceleration onset rate curve). For the entire group, the onset rate for the exposures ranged from 0.05 to 7.6 G/s, the LOCINDTI ranged from 5 to 106 s, and the +Gz level ranged from +2.5 to +11.7 Gz. The resulting curves do not simply represent relaxed G-LOC tolerance; all types of exposures, including those with and without performance of an AGSM and/or wearing an AGS composed the dataset. +Gz level tolerance is characterized by the contribution of all aspects of protection existing during an exposure. Available data did not cover the broad range of GORs necessary to thoroughly describe +Gz level tolerance.

The starting point in developing the specific G-LOC curves was based on previously established +Gz-time tolerance curves and physiologic principles defining tolerance [7-10,17,19,24-26]. These suggested that the current dataset would produce hyperbolic curves. Based on these assumptions, the vertical asymptote represents the minimum time for LOCINDTI in each curve and should reflect the value of the FBP. The horizontal asymptotes differ in the two curves representing minimum +Gz level tolerance in one (Figure 1) and the *x*-axis in the other (Figure 2). The asymptotes and arms of the curves provide the limiting G-LOC values for the vertical and horizontal aspects of the G-LOC curves and were analyzed separately before integrating them into the final two curves.

### ROR data analysis (the vertical asymptote)

#### The maximum +Gz level curve

The ROR-specific dataset taken from the left side of Figure 1 representing 406 G-LOC episodes (+2.5 to +9 Gz) reflects the neurologic tolerance aspect of the human response to ROR acceleration as a function of the maximum +Gz level reached during an exposure (GMAX). LOCINDTI had a mean of 10.01 ± 3.32 s, 5 s minimum, 33 s maximum, range of 28 s, and a median of 9 s. The 95% confidence level (CL) for the mean LOCINDTI was 0.32 s (9.69 s to 10.34 s confidence interval). ANOVA results indicated that the mean LOCINDTI did not decrease in duration when the +Gz level was above +7 Gz. Table 1 provides the descriptive statistics for LOCINDTI as a function of +Gz level. The transitional band, where LOCINDTI gradually lengthens, defines the shape of the curve as it changes from rapid to gradual types of acceleration stress with the mean LOCINDTI increasing from 10.61 ± 4.59 s for the +6 to <+7 Gz level through 17.13 ± 7.26 s for the +2.5 to <+4.0 Gz level. The transitional range of the curve was obvious over the +2.5 to <+6 Gz band. The LOCINDTIs at levels +2.5 to <+4 Gz were significantly longer than those at levels +4 Gz and above (*F* (4, 401) = 13.67, *p* < 0.0001; HSD = 3.97, *p* < 0.01). Therefore, the ROR data above +7.0 Gz were considered to represent the vertical arm and *y*-asymptote of this curve. The vertical arm of this G-LOC curve was considered to have become equivalent to its asymptote. The mean LOCINDTI value for this data set of 9.65 ± 2.66 s (minimum 5 s, maximum 22 s, median 9 s, and 95% CL 0.30) defines the mean curve's asymptote, and 5 s defines the minimum curve asymptote.

**Table 1:** **Relationship of LOCINDTI and specific +Gz levels (GMAX) for the ROR dataset of Figure **1

**GMAX interval ROR dataset**	**Loss of consciousness induction time (s)**							
**+Gz**	***N***	**Min**	**Max**	**Range**	**Mean**	**SD**	**SE**	**Median**
9	164	6	20	14	9.20	2.60	0.2	9
8 to <9	97	5	18	13	9.00	2.15	0.22	9
7 to <8	70	5	22	17	9.64	3.29	0.39	9
6 to <7	49	5	33	28	10.61	4.59	0.66	10
5 to <6	8	6	17	11	11.5	4.21	1.49	11.5
4 to <5	10	6	15	9	12.3	2.95	0.93	13.5
2.5 to <4	8	12	32	20	17.13	7.26	2.57	14
≥7	331	5	22	17	9.65	2.66	0.15	9
All	406	5	33	28	10.01	3.32	0.16	9

#### Acceleration onset rate curve

Table 2 provides the descriptive statistics for the ROR dataset taken from the left side of Figure 2 representing 432 G-LOC episodes. This portion of the overall dataset reflects LOCINDTI versus onset rates of up to 4 G/s and represents the neurologic tolerance-limiting aspect of the physiologic response to acceleration. The LOCINDTI had a mean of 9.58 ± 3.03 s (5 s minimum, 32 s maximum, 27 s range, 9 s median, and 95% CL of 0.286). No G-LOC episode had a LOCINDTI less than 5 s. There was no decrease in LOCINDTI as the onset rate increased from 1 to 7.6 G/s. While the ANOVA *F* (3,387) = 5.22, indicated a *p* < 0.001, the post-hoc analysis did not demonstrate a significant statistical difference among these onset rates (HSD_0.05_ = 1.67). The human neurologic tolerance to rapid onset acceleration for exposures 1.0 G/s or higher had a mean value of 9.10 ± 2.4 s, with a minimum value of 5 s and a maximum value of 20 s. The 95% confidence interval for the mean LOCINDTI was 0.24 s (8.86 to 9.34 s). There was a trend for LOCINDTI to increase for onset rates below 1.0 G/s as shown in Table 2. Multiple comparison analysis of LOCINDTI by onset rate in bands between 0.2 and <1 G/s was performed with no statistically significant differences among the onset rates (*F* (3,37) = 2.32, *p* < 0.091). There was a statistically significant difference (*t* = 2.02, *p* = 5.57 E-09) between the bands 0.2 to <1 G/s (14.12 ± 4.37 s) and ≥1 G/s (9.10 ± 2.40 s). The data below 1.0 G/s were considered to represent a transitional band (between rapid and gradual onset rates). The ROR data at and above 1.0 G/s were considered to represent the vertical arm of this G-LOC curve since the LOCINDTI values did not significantly differ at or above 1.0 G/s. The vertical arm of the G-LOC curve was therefore considered to have become equivalent to the *y*-asymptote defining the neurologic tolerance with a value of 9.10 ± 2.4 s.

**Table 2:** **Relationship of LOCINDTI and specific onset rate intervals for the ROR dataset of Figure **2

**Onset rate interval ROR dataset**	**Loss of consciousness induction time (s)**							
**G/s**	***N***	**Min**	**Max**	**Range**	**Mean**	**SD**	**SE**	**Median**
≥4	8	5	14	9	9.13	3.14	1.11	8.5
3 to <4	120	5	20	15	8.83	2.52	0.23	8
2 to <3	129	5	16	11	8.68	2.18	0.19	8
1 to <2	134	5	20	15	9.75	2.35	0.20	9
0.5 to <1	25	6	21	15	13.08	3.37	0.67	12
0.2 to <1	41	6	32	26	14.12	4.37	0.68	13
0.7 to <1	6	9	15	6	11.33	2.42	0.99	11
0.6 to <0.7	10	6	20	14	12.5	3.63	1.15	12
0.5 to <0.6	9	12	21	9	14.89	3.02	1.01	14
0.2 to <0.5	16	8	32	24	15.75	5.29	1.32	14.5
≥1	391	5	20	15	9.10	2.40	0.12	9
All	432	5	32	27	9.58	3.03	0.15	9

### GOR data analysis (the horizontal asymptote)

#### The maximum +Gz level curve

**Figure 3:**
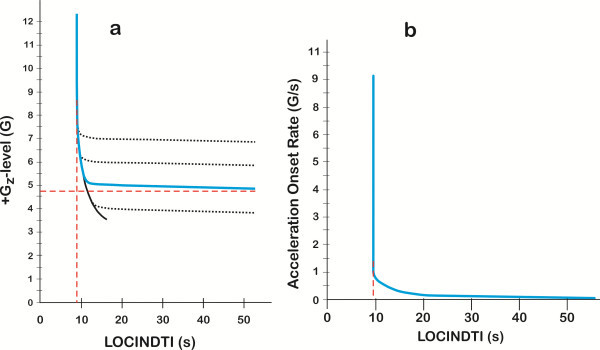
**G-LOC curves for +Gz level and acceleration onset rate as functions of LOCINDTI.** +Gz level (**a**) and acceleration onset rate (**b**). Asymptotes are shown as dashed lines. The three dotted line horizontal arm tolerances in (**a**) represent the characteristics of how ranges of different population +Gz level tolerances integrate with the vertical neurologic tolerance arm of the curve.

The GOR specific dataset within Figure 1, consisting of 293 G-LOC episodes, revealed three groups of G-LOC responses indicating three main onset rates used in the exposures: 0.064 ± 0.004 (*n* = 48), 0.086 ± 0.003 (*n* = 186), and 0.105 ± 0.06 (*n* = 54) G/s. The range of +Gz levels for G-LOC occurrence encompassing the entire GOR group (*n* = 293) was +3.6 to +11.7 Gz. Data analysis included runs ≤ +9 Gz (*n* = 293). Descriptive statistics for the GOR dataset are shown in Table 3. The mean G-LOC +Gz level for the entire GOR dataset was +7.31 ± 1.32 Gz (*n* = 293, median = +7.6 Gz). The minimum +Gz level threshold tolerance (+4.7 Gz) for the entire GOR group was defined as −2 standard deviations below the overall mean of +7.31 Gz. The horizontal arm of the curve was constructed by connecting the curve defining the minimum of the exhibited vertical arm transition occurring between +5 Gz and +6 Gz with the minimum +Gz level threshold tolerance for the entire group (+4.7 Gz). The horizontal arm therefore represents the minimum threshold +Gz level tolerance curve for this population. The asymptote to the minimum +Gz level-threshold tolerance curve was then constructed parallel to the *x*-axis as shown in Figure 3a (dashed horizontal line). The minimum threshold +Gz level-G-LOC curve should approach the horizontal asymptote as LOCINDTI increases. +Gz level tolerance to GOR exposures is well known to have considerable variability. In addition, since the exposures included relaxed, unprotected along with fully protected exposures, it was not surprising that the +Gz level range was very broad.

**Table 3:** **Relationship of LOCINDTI and specific intervals of +Gz level tolerance for GOR dataset of Figure **1

**GMAX interval GOR dataset**	**Loss of consciousness induction time (s)**							
**+Gz**	***N***	**Min**	**Max**	**Range**	**Mean**	**SD**	**SE**	**Median**
9	19	93	93	11	92.63	2.79	0.64	93
8 to < 9	98	65	95	30	85.06	6.47	0.65	86
7 to < 8	68	56	106	50	74.51	11.26	1.37	76
6 to < 7	55	48	96	48	67.51	12.31	1.66	65
5 to < 6	40	36	78	42	57.78	13.96	2.21	57.5
3.6 to 5	13	38	65	27	53	9.56	2.65	56
All	293	36	106	70	74.66	15.11	0.88	77

#### Acceleration onset rate curve

For GOR exposures with onset rates between 0.05 and <0.2 G/s in Figure 3b, the tolerance time ranged from 36 to 106 s (*n* = 298, mean 74.41 ± 15.11 s, median 77 s, and a 95% CL of 1.72 s). This G-LOC curve's horizontal arm approaches its asymptote, the *x*-axis, as onset rates decrease. The distribution of LOCINDTI from 36 to 106 s reflects the distribution of tolerances based on how long it takes to reach +Gz tolerance. A longer LOCINDTI reflects ‘longer’ tolerance for a given onset rate. The slowest onset rate group, that utilized an onset rate of approximately 0.06 G/s, had the lowest level for G-LOC occurrence and longer LOCINDTI at a given G-LOC level. A complete analysis of the GOR dataset is beyond the scope of this analysis.

### The integrated maximum +Gz level curve

**Figure 4:**
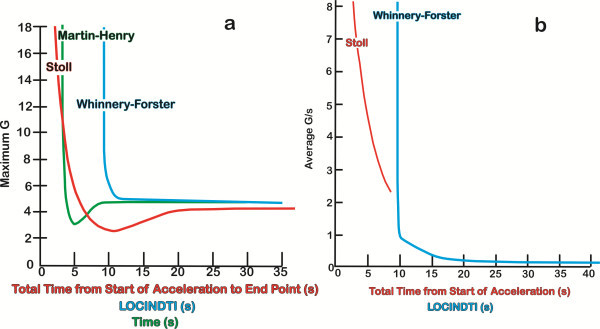
**Comparison of the existing G-LOC tolerance curves. **Comparison of the existing G-LOC tolerance curves for (**a**) +Gz level tolerance comparisons between Stoll (red), Martin-Henry (green), and the current Whinnery-Forster (blue) curves as a function of the loss of consciousness induction time and (**b**) acceleration onset rate (G/s) as a function of total time from start of acceleration (loss of consciousness induction time) between Stoll and the current Whinnery-Forster curve (curves redrawn from the original manuscripts, with the Martin-Henry curves converted from a logarithmic to linear scale).

The final single line, population mean +Gz level versus LOCINDTI curve is shown in Figures 3a and 4a. This G-LOC curve defines the mean neurologic tolerance associated with an ROR response to acceleration stress. The mean of the curve for ROR exposures was 9.65 s. The vertical arm of the curve becomes equivalent to the vertical asymptote of the hyperbola. As the onset rates decrease from ROR to GOR, the vertical arm of the curve transitions into the established level of G-LOC occurrence as defined by the GOR data forming the horizontal arm of the curve. The GOR portion of the curve very gradually approaches the horizontal +Gz level tolerance (*x*-asymptote) just below +4.7 Gz (minimum tolerance threshold) as onset rates decrease. For a population with different +Gz level tolerances, the shape of the transition from ROR to GOR exposures in the +4 Gz through +7 Gz range can be predicted as shown in Figure 3a. The shape of the transition curve over this range was based on the LOCINDTI transitional times of 10.61 to 17.13 s. Continuous with the transitional +Gz levels the G-LOC curve, the horizontal aspect of the curve approaches the horizontal +Gz level tolerance asymptote over the period of time that protected +Gz level tolerance can be maintained. All of the curves would be predicted to have a characteristically sharp transition from ROR to GOR tolerance over a small, transitional +Gz level range. For the entire GOR group (≤0.2 G/s), G-LOC occurs in a mean time of 74.41 s. This value for LOCINDTI is only useful in a general sense to describe the longer overall time of exposure associated with GOR in comparison to ROR. The predicted time to reach the minimum +Gz level threshold tolerance for a specific gradual onset rate should be calculated specifically for each gradual onset rate profile.

### The integrated acceleration onset curve

With the characterization of the neurologic tolerance gained from the ROR dataset and the onset rates representing those utilized for GOR exposures, the two halves of the acceleration onset rate curve were integrated based on hyperbolic curve geometry. The shape of the curve as it changes from ROR to GOR exposures was defined by G-LOC data for transitional onset rates. The ROR portion of the acceleration onset rate curve has a value of 9.10 s. It should be noted that the vertical arm of this hyperbolic curve has become equivalent to its asymptote. The transitional onset rates from <1.0 to 0.2 G/s defined the curve segment between the ROR and GOR datasets. The curve then gradually decreases within the GOR range to the lowest experimentally utilized onset rate value of 0.05 G/s and would thereafter be predicted to approach the horizontal asymptote represented by the *x*-axis. Taking the mean responses only, a single line curve representing the final acceleration onset rate versus LOCINDTI curve is shown in Figures 3b and 4b.

## Discussion

The two G-LOC curves represent the response to the extreme acceleration environment that exceeds human tolerance resulting in complete LOC in healthy humans. The primary goal of this investigation was the development of the G-LOC component of previously developed general acceleration time-tolerance curves. These earlier general tolerance curves were based on subject-reported visual symptoms and scanty G-LOC data over a less than complete +Gz exposure range [19,26]. The two G-LOC curves developed represent new underlying physiological aspects of acceleration tolerance, significantly different shape and time relationships, and an improved data-driven standard for human exposure to the environmental exposure limit of healthy humans to acceleration stress. The curve separates two dramatically different neurophysiological states, the human state of consciousness from the state of unconsciousness. The G-LOC curves represent progress toward curves that may apply to any type of acceleration profile for all individual conditions and not limited simply to laboratory-type profiles in relaxed subjects as previously existed [19,26]. A specific study aim was to quantify the acceleration envelope associated with G-LOC, utilizing acceleration stress parameters (onset rates and +Gz levels) readily obtainable from operational flight profiles. The newly developed G-LOC curves, based on 888 G-LOC episodes, have applicability to accident or incident reconstruction involving possible in-flight G-LOC. The healthy population from which these G-LOC episodes were obtained was predominately fighter pilots [13], which have been suggested to be a requirement for operational +Gz tolerance studies [27]. The availability of a database, requiring years to acquire 888 G-LOC episodes in healthy humans, provided the information necessary for the development of more complete operationally oriented G-LOC curves.

**Figure 5:**
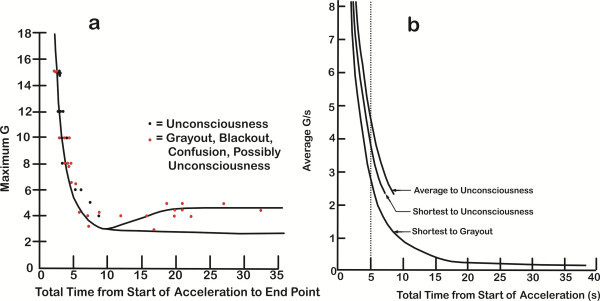
**Original stoll curves. **Redrawn from the original stoll curves (**a**) with maximum +Gz level versus time from start of acceleration to endpoints (unconsciousness, visual symptoms, and possible unconsciousness) and (**b**) average onset rate versus time from start of acceleration.

Previous curves were developed to provide a description of less extreme environments associated with relaxed tolerance to specific types of acceleration profiles predominately on the basis of visual symptoms and a minimal number of G-LOC episodes as illustrated by the Stoll curves presented in Figure 5a, b [19]. In addition, because of the concerns related to cardiovascular reflex response as a function of time, the previous curves were only applicable to profiles starting from resting conditions. The G-LOC curves differ in requiring only the definition of the profile that leads to exceeding +Gz level tolerance resulting in the G-LOC episode. The new curves, which had the majority of G-LOC episodes, result despite maximum, all-out muscular straining, and proficient anti-G respiratory maneuvers being performed by healthy individuals to avoid LOC.

Comparison with previous +Gz-time tolerance curves revealed that the slope of the vertical arm of the current G-LOC curves is in agreement with the Gauer [7], Martin-Henry [26], and Gauer-Henry [8-10] curves. The current curves do not support the curve subsequently developed by Stoll in terms of the shape (slope) of the vertical arms or in the time to G-LOC, as shown in Figure three of that publication [19]. The current +Gz level curve and the Martin-Henry curves have vertical arms that do not approach the *y*-axis (infinite slope) above +7 Gz, indicating that at this +Gz level, tolerance becomes independent of the +Gz level of exposure. Our results also support the tolerance curve modeling of Moore et al. [20] and Hrebien [28] who also pointed out inconsistencies with the Stoll curve that included joining data points representing visual symptoms with LOC data points, especially in the GOR range. Visual symptoms are well known to occur at lower +Gz levels than the limiting +Gz level that induces G-LOC in the GOR range. Figure 4 also reveals that the current curve differs in the time to onset of G-LOC for ROR exposures in comparison to all earlier curves. The Stoll curve measured the time from base +Gz to LOC just as the current Whinnery-Forster curve does, which was defined as LOCINDTI. The Martin-Henry curve however labeled it as ‘time’ which was the time at maximum +Gz rather than the time from the onset of +Gz stress. Both of the newly developed curves indicate that time is four to five times longer than that indicated by the Stoll curve. The current curves are in much closer agreement with the later Kydd-Stoll primate curve [25] in terms of shape and time to LOC.

The mean LOCINDTI from the vertical arm (ROR) of the current +Gz level versus time G-LOC curve above +5 Gz was 9.70 s. The LOCINDTI values for the Stoll curve above +5 Gz decreased from 5 to 2 s as the +Gz level increased. These curve values may be compared with the equivalent LOCINDTI from other studies that rapidly induce LOC. Rossen acutely arrested cerebral circulation in 74 humans using a cervical pressure cuff with the mean time of 6.41-6.91 s [29,30]. The mean time from vascular neck restraint (used by police to control combative individuals) to LOC defined by onset of eye fixation in 24 healthy volunteer police officers was 7.9 s (range 6.3 to 12.2 s) [31]. Sauvageau et al. reported that the time to loss of consciousness in 14 filmed human hangings is 10.4 ± 3.0 s (range 8 to 18 s) [32]. The mean LOCINDTI values from completely unprotected (no anti-G straining maneuver or anti-G suit) G-LOC episodes reported by Houghton et al. for two types of ROR runs (onset rates (1.0-1.5 G/s and 2.2 G/s – 3.0 G/s) to a mean of +6.1 Gz were 9.2 and 10.7 s [33]. The mean LOCINDTI was 12.65 s in the large US Navy Pensacola study, taking 935 individuals to G-LOC with a mean +Gz level of +5.3 Gz and a mean onset rate of 0.8 G/s [34]. Even the limiting time to complete visual blackout with abrupt onset of rapidly applied external eye pressure equal to or greater than systolic ophthalmic artery pressure in 10 normal individuals was 9.40 ± 4.10 s [35]. None of these reported values are 5 s or less as defined by the Stoll curve. All these values are compatible with the currently developed Whinnery-Forster G-LOC curves.

Previous +Gz-time tolerance was represented by a single line or single lines (or band) for each sign and symptom. No indication of the variability or range was associated with any of these curves. For the Stoll curves (Figure 5a, b), only 14 G-LOCs were presented on the left, rapid onset aspect of the curve; this limitation posed a major hindrance in refining a complete curve and its components. The current G-LOC curves on the other hand provide a population standard for healthy human response to acceleration stress. Comparison of the main characteristics of the Stoll and Whinnery-Forster curves is provided in Table 4. Although the differences may appear small, in the extreme environment of the combat fighter pilot, they are very significant. The acceleration onset rate curve also provides the opportunity to define acceleration onset rate on the basis of the neurophysiologic response to G-LOC. All onset rates above 1.0 G/s should be considered rapid onset (ROR) based on the onset rate that produces immediate dependence on neurologic (CPNS) tolerance before G-LOC occurs, with LOCINDTI being constant. Transitional onset rates are those between <1.0 and 0.2 G/s, where the LOCINDTI begins to increase. Gradual onset (below 0.2 G/s) is based on rates that do not cause tolerance to become rapidly dependent only on neurologic (CPNS) tolerance. This analysis sets the stage for further development of the mathematical equations defining the G-LOC hyperbolic curves.

**Table 4:** Acceleration onset rate versus LOCINDTI curve comparison between Stoll and Whinnery-Forster curves

**Onset rate vs. LOCINDTI curve characteristics**	**Stoll**	**Whinnery-Forster**
*y*-Asymptote	2.0 to 2.5 s	No asymptote (curve value constant at 9.10 s)
*x*-Asymptote	1.0 G/s	*x*-axis
Transitional onset rate range	Continuous decrease as onset rate increases approximately 3 to 1 G/s	<1.0 to 0.2 G/s
5 G/s: LOCINDTI	5 s	9.13 s
2 G/s: LOCINDTI	12 s	8.68 s
1 G/s: LOCINDTI	Considered GOR	9.75 s
Ranges provided for G-LOC	No (single line G-LOC curve)	Yes (G-LOC curve with associated descriptive statistics)
Symptoms included	Yes (visual loss)	No (G-LOC only)
Number of endpoints	40 (14 LOC)	888 LOC
Subject status	Relaxed from rest	Both relaxed and protected

## Conclusions

The G-LOC curves provide an improved standard for acceleration responses that result from exposure to profiles with GORs of 0.05 G/s through RORs 7.6 G/s and +Gz levels from +2.5 to +11.7 Gz. The minimum LOCINDTI for G-LOC for the population (*N* = 888 G-LOC episodes) was 5 s. In addition, the mean ROR LOCINDTIs of 9.10 and 9.65 s were not found to decrease as onset rates increased above 1.0 G/s or as +Gz levels increased above 7. The G-LOC response becomes constant such that any exposure above +Gz tolerance becomes immediately dependent on CPNS tolerance to +Gz-induced ischemia. These times for rapid induction of CPNS ischemia are important in aerospace medicine and provide a diagnostic comparison when evaluating individuals with transient loss of consciousness in clinical medicine. It should be emphasized that G-LOC episodes were observed at 5 s, and to minimize in-flight risk, a timely and proficient anti-G straining maneuver is essential for all high-+Gz exposures.

## Abbreviations

GOR: Gradual onset rate/run; ROR: Rapid onset rate/run; LOC: Loss of consciousness; G-LOC: +Gz-induced loss of consciousness; GMAX: maximum +Gz level; LOCINDTI: Loss of consciousness induction time; FBP: Functional buffer period; CPNS: Cephalic nervous system.

## Competing interests

The authors declare that they have no competing interests.

## Authors' contributions

TW was responsible for the data assembly, development, and analysis while participating in the statistical analysis and in drafting the manuscript. EMF conceived of the study, was responsible for the initial data collection and the statistical analysis while participating in the analysis and in drafting the manuscript. Both authors contributed to, read, and approved the final manuscript.
